# Sibjotang Protects against Cardiac Hypertrophy In Vitro and In Vivo

**DOI:** 10.3390/life13122307

**Published:** 2023-12-07

**Authors:** Chan-Ok Son, Mi-Hyeon Hong, Hye-Yoom Kim, Byung-Hyuk Han, Chang-Seob Seo, Ho-Sub Lee, Jung-Joo Yoon, Dae-Gill Kang

**Affiliations:** 1 Department of Ophthalmology, Konkuk University School of Medicine, Gwangjin-gu, Seoul 05030, Republic of Korea; study0815@naver.com; 2 Hanbang Cardio-Renal Syndrome Research Center, Wonkwang University, 460, Iksan-daero, Iksan, Jeonbuk 54538, Republic of Korea; mihyeon123@naver.com (M.-H.H.); hyeyoomc@naver.com (H.-Y.K.); arum0924@naver.com (B.-H.H.); host@wku.ac.kr (H.-S.L.); 3 KM Science Research Division, Korea Institute of Oriental Medicine, Daejeon 34054, Republic of Korea; csseo0914@kiom.re.kr; 4 College of Oriental Medicine, Wonkwang University, 460, Iksan-daero, Iksan, Jeonbuk 54538, Republic of Korea

**Keywords:** heart failure, heart diseases, apoptosis, H9c2 cell, isoproterenol

## Abstract

Cardiac hypertrophy is developed by various diseases such as myocardial infarction, valve diseases, hypertension, and aortic stenosis. Sibjotang (十棗湯, Shizaotang, SJT), a classic formula in Korean traditional medicine, has been shown to modulate the equilibrium of body fluids and blood pressure. This research study sought to explore the impact and underlying process of Sibjotang on cardiotoxicity induced by DOX in H9c2 cells. In vitro, H9c2 cells were induced by DOX (1 μM) in the presence or absence of SJT (1–5 μg/mL) and incubated for 24 h. In vivo, SJT was administrated to isoproterenol (ISO)-induced cardiac hypertrophy mice (*n* = 8) at 100 mg/kg/day concentrations. Immunofluorescence staining revealed that SJT mitigated the enlargement of H9c2 cells caused by DOX in a dose-dependent way. Using SJT as a pretreatment notably suppressed the rise in cardiac hypertrophic marker levels induced by DOX. SJT inhibited the DOX-induced ERK1/2 and p38 MAPK signaling pathways. In addition, SJT significantly decreased the expression of the hypertrophy-associated transcription factor GATA binding factor 4 (GATA 4) induced by DOX. SJT also decreased hypertrophy-associated calcineurin and NFAT protein levels. Pretreatment with SJT significantly attenuated DOX-induced apoptosis-associated proteins such as Bax, caspase-3, and caspase-9 without affecting cell viability. In addition, the results of the in vivo study indicated that SJT significantly reduced the left ventricle/body weight ratio level. Administration of SJT reduced the expression of hypertrophy markers, such as ANP and BNP. These results suggest that SJT attenuates cardiac hypertrophy and heart failure induced by DOX or ISO through the inhibition of the calcineurin/NFAT/GATA4 pathway. Therefore, SJT may be a potential treatment for the prevention and treatment of cardiac hypertrophy that leads to heart failure.

## 1. Introduction

Heart failure is a disease caused by insufficient blood supply to the body due to cardiac dysfunction and is well known as a leading cause of morbidity and mortality worldwide [1]. Cardiac remodeling has characteristics of heart failure, including myocardial hypertrophy, fibrosis, and progressive ventricular expansion [2]. Cardiac hypertrophy is developed by various diseases such as myocardial infarction, valve diseases, hypertension, and aortic stenosis [3]. Cardiac hypertrophy is well known for its characteristics including the thickening of heart muscles and is associated with chronic heart failure [4]. It is established that hypertrophy and apoptosis in cardiomyocytes occur in the progression of heart failure and arrhythmias [3]. Therefore, myocardial hypertrophy is one of the most frequent causes of heart failure and is a critical sign of a negative prognosis.

Doxorubicin (DOX), the most potent and widely used anthracycline, has been used to treat malignancies like leukemias, lymphomas, soft tissue sarcomas, and other solid tumors [5]. Anthracyclines have been known to induce cancer cell death by a variety of mechanisms including inhibition of the topoisomerase II enzyme, damage to cell membranes, and generation of free radicals [6,7,8]. In addition, anthracyclines have been shown to modulate various signaling pathways including the apoptosis pathway [9]. However, the anti-neoplastic application of DOX is limited by its side effects, which result in cardiac hypertrophy, fibrosis, and cell death [10,11,12]. DOX-induced cardiotoxicity has been attributed to apoptosis, oxidative stress, mitochondrial impairment, DNA strand breaks, sarcomere structural alterations, and calcium overloading [13,14].

In the mammalian heart, cardiomyocytes and cardiac fibroblasts account for 90% of the cells in the myocardium. Cardiomyocytes are key cells not only in the maintenance of cardiac structure but also in the contraction function of heart. The contraction function of heart is associated with cytoskeletal structures, which act in myosin-based contractile systems [15,16]. Stress fibers, a key component of cytoskeletal structures, are seen in almost all prominent cardiovascular disorders such as hypertension, cardiac fibrosis, cardiomyopathy, valvular heart diseases, and heart failure [17]. Stress fibers are contracted under stress and are composed of a filamentous actin (F-actin). In addition, it is well established that stress fiber formation is discovered in cardiac hypertrophy [18].

Atrial natriuretic peptide (ANP) and brain natriuretic peptide (BNP) are major hormones produced by the heart, and these hormones play a role in regulating the heart and blood pressure [19,20]. Previous studies have demonstrated the clinical utility of ANP and BNP in the assessment of the severity of heart failure, particularly left ventricular systolic dysfunction [21,22]. Functionally distinct, the two types of cardiac MHC isoforms, alpha-myosin heavy chain (α-MHC) and beta-myosin heavy chain (β-MHC), are expressed influenced by developmental processes and hormonal factors [23]. The expression ratio of these two isoforms can vary in conditions like heart failure or cardiac hypertrophy [24]. The increased expression of β-MHC can serve as an early sensitive indicator of cardiac hypertrophic response [25]. Myosin light chain-2v (MLC-2v), a ventricular myosin light chain, is essential for the formation of thick filaments in heart muscle cells and is linked to inherited hypertrophic cardiomyopathy [26,27].

MAPK (Mitogen-Activated Protein Kinase) is an important kinase family responsible for mediating various signals within cells. Among this family, ERK1/2 (Extracellular Signal-Regulated Kinase 1/2) primarily plays a role in transmitting signals related to cell growth, division, and survival. It was established that the ERK1/2 MAPK pathways participate in hypertrophic signaling in cardiac myocytes [28,29]. ERK1/2 MAPK is stimulated by diverse neurohumoral triggers and by the stretching of cardiac myocytes, both in cultured cells and in vivo [30]. Previous studies have demonstrated that ERK1/2 also plays a role in the regulation of GATA4 phosphorylation, a transcription factor [31]. In the heart, p38 MAPK is a part of a crucial signaling pathway involved in cellular stress responses and inflammation reactions. p38 MAPK is activated by various external signals, such as cellular stress, cytokines, and cardiac damage. Activation of p38 MAPKs is necessary for the binding of GATA4 to the BNP gene induced by hypertrophic agonists and is sufficient for GATA-dependent BNP gene expression [31,32]. GATA4, a transcription factor, serves as a key regulator of cardiac genes in normal cardiac development and pathological hypertrophy [33]. In cardiac myocytes, GATA4 is primarily phosphorylated in response to agonist stimulation through the MEK1-ERK1/2 pathway, while the JNK1/2 or p38 MAPK pathways have weaker effects [34]. Furthermore, GATA4 controls the expression of various genes, encompassing α-MHC, β-MHC, cardiac troponin-C, ANP, and BNP [35]. Excessive calcium accumulation increases the activation of calcineurin which also dephosphorylates the nuclear factor of activated T cells (NFAT) and triggers its translocation from the cytosol to the nucleus causing hypertrophy [36,37]. The nuclear factor of activated T cells (NFAT) is a transcription factor family that mediates various intracellular signaling pathways. The NFAT family comprises several members (NFAT1, NFAT2, NFAT3, NFAT4, and NFAT5), each having specific functions in different cell types and tissues. NFAT is primarily activated through pathways associated with calcium signaling and plays crucial roles in various cellular functions, notably in cell activation, differentiation, and survival [36]. Among them, NFAT3 (NFATc4) is expressed especially in the heart. The Calcineurin–NFAT (Calcineurin–Nuclear Factor of Activated T-cells) pathway is one of the primary regulatory mechanisms for cardiac hypertrophy, modulating the expression of specific genes that influence the size and function of cardiac cells. In particular, the activation of this pathway promotes the increased expression of cardiac hypertrophy-related genes such as SKA (skeletal α-actin), β-MHC, and BNP [38,39].

In response to apoptotic stimuli, pro-apoptotic members of the Bax and the Bcl-2 family are activated on the mitochondria to induce the release of cytochrome c. Released cytochrome c results in the formation of the apoptosome and the apoptosome activates caspase-9, which leads to the activation of caspase-3. This process leads to the same type of apoptotic response as observed for the extrinsic pathway [40,41,42].

In traditional Korean medicine, various herbal prescriptions have been used for the treatment of heart diseases. Sibjotang (SJT, Shizaotang in Chinese, Jyusoto in Japanese) was recorded in a traditional Chinese medical book named “*Shanghan Lun*” and a traditional Korean medical book named “*Donguibogam*”. SJT has been recorded to be used extensively for symptoms accompanied by edema. SJT is composed of four component herbal medicines: *Euphorbia kansui*, *Euphorbia pekinensis*, *Daphne genkwa*, and *Ziziphus jujube*. Recently, SJT has been known to have anti-inflammatory, anti-tumor, and anti-allergy effects [43]. In a previous study, compounds of SJT were identified as salvianolic acid B, rosmarinic acid, apigenin 7-*O*-β-glucuronide, apigenin, and yankanin by analysis of 1D and 2D NMR. Additionally, it is reported that SJT increased the positive inotropic effect in rabbit atria [44]. However, research on whether SJT improves cardiac dysfunction caused by cardiac hypertrophy has not yet been conducted. This study investigated the protective effect of SJT on cardiac hypertrophy through the regulation of the calcineurin/NFAT/GATA4 pathway.

## 2. Materials and Methods

### 2.1. Chemicals and Materials

Dulbecco’s Modified Eagle Medium (DMEM), fetal bovine serum (FBS), 0.05% trypsin-EDTA, antibiotic-antimycotic, and Alexa Fluor™ 488 Phalloidin (A12379) were purchased from Thermo Fisher Scientific (Waltham, MA, USA). Doxorubicin (sc-280681), primary antibody p38 (1:1000; sc-7972), ERK1/2 (1:1000; sc-135900), p-ERK1/2 (1:1000; sc-7383), p-GATA4 (1:500; sc-377543), Bcl-2 (1:500; sc-7382), Lamin B1 (1:2000; sc-374015), and β-actin (1:2000; sc-47778) were purchased from Santa Cruz Biotechnology (Dallas, TX, USA); p-p38 (1:1000; #9211), JNK (1:1000; #9252), p-JNK (1:1000; #9251), NFAT3 (1:1000; #2188), Caspase-9 (1:1000; #9502), Caspase-3 (1:1000; #9665), Bax (1:1000; #2772), and α-tubulin (1:1000; #2144) were purchased from Cell signaling technology (Danvers, MA, USA); Calcineurin (1:1000; 610259) was purchased from BD Bioscience (San Jose, CA, USA).

### 2.2. Preparation of Sibjotang

SJT consists of four herbs, *Euphorbia kansui*, *Euphorbia pekinensis*, *Daphne genkwa*, and *Ziziphus jujube*. The botanical ingredients *Euphorbia kansui*, *Euphorbia pekinensis*, *Daphne genkwa*, and *Ziziphus jujube* were sourced from the Herbal Medicine Cooperative Association in Iksan, Jeonbuk, Korea. Reference samples of SJT, labeled as HBG132, were cataloged and stored at the Herbarium of the Professional Graduate School of Oriental Medicine at Wonkwang University, located in Iksan, Korea. The manufacturing method follows the regimen based on a Sanghanlun scale. *Euphorbia kansui*, *Euphorbia pekinensis*, and *Daphne genkwa* were scored the same weight of 37.5 g and *Ziziphus jujube* was scored as 85 g. The dried pre-SJT herb (500 g) was subjected to extraction using 2000 mL of distilled water at a temperature of 100 °C for a duration of 4 h. Post-extraction, the mixture was passed through Whatman No. 5 filter paper and later centrifuged at a speed of 3000 rpm for a period of 10 min at a temperature of 4 °C. The supernatant was then condensed using a rotary vacuum evaporator (Model N-11, manufactured by Tokyo Rikakikai, Tokyo, Japan). Finally, the concentrated extract was freeze-dried using a lyophilizer (PVTFD10RS, IlsinBioBase, Yangju, Republic of Korea) and retained at −70 °C until required. The powdered form of Sibjotang was dissolved in PBS at a 1000-fold higher concentration before being used in in vitro experiments and prepared as a stock solution. For instance, to achieve a final concentration of 5 μg/mL for STJ treatment, we prepared a stock solution of SJT with a concentration of 5 mg/mL.

### 2.3. HPLC Fingerprinting Analysis of SJT

HPLC fingerprinting analysis of the SJT and its authentic standards (apigenin, apigenin 7-*O*-β-glucuronide, rosmarinic acid, and salvianolic acid B) was conducted using a Shimadzu Prominence LC-20A series system (Shimadzu Co., Kyoto, Japan) coupled with a photodiode array (PDA) detector and an analytical column (SunFire C18, 250 × 4.6 mm, 5 μm, Waters, Milford, MA, USA). The SJT and its standard solution were analyzed using a sequential gradient mobile phase system of 0.1% (*v*/*v*) aqueous formic acid (mobile phase A) and 0.1% (*v*/*v*) formic acid in acetonitrile (mobile phase B), namely, 10% B (initial), 50% B (25 min; hold for 5 min), and 10% B (40 min; hold for 10 min). The flow rate was 1.0 mL/min and the injection volume was 10.0 μL. The column thermostat and auto-sampler were maintained at 40 °C and room temperature, respectively. The chromatographic data were processed by the LabSolutions software (version 5.117, Shimadzu Co.). Each standard stock solution for quantitative determination was prepared at 1.0 mg/mL using methanol. The SJT solution for quantitative analysis was dissolved in 70% (*v*/*v*) methanol at a concentration of 100 mg/10 mL and then subjected to ultrasonic extraction for 1 h. To quantify two components (apigenin 7-*O*-β-glucuronide and salvianolic acid B), the prepared SJT solution was diluted 10-fold and then analyzed.

### 2.4. Cell Culture

The rat-derived H9c2 cardiomyocytes (CRL-1446) were purchased from the American Type Culture Collection (Manassas, VI, USA). Cells were cultured in DMEM supplemented with 1% penicillin-streptomycin antibiotic-antimycotic mixture, 2 mM glutamine, 1.5 g/L sodium bicarbonate, 3.5 g/L glucose, and 10–15% fetal bovine serum (FBS). Culturing was maintained in a humidified incubator at 37 °C with 95% air and 5% CO. When cell confluence reached approximately 80–90%, cells were detached using Trypsin-EDTA solution, and the culture medium was replaced every three days.

### 2.5. Cell Viability Assay

Cytotoxicity was assessed using the 3-(4,5-dimethylthiazol-2-yl)-2,5-diphenyltetrazolium bromide (MTT) assay. H9c2 cells were seeded in a 96-well culture plate at a density of 1 × 10^4^ cells per well. They were then cultured in serum-free DMEM with various concentrations of SJT for 24 h. Subsequently, 10 μL of MTT solution (0.5 mg/mL) was added to each well, and the plates were incubated for an additional 4 h at 37 °C. After that, the MTT solution was removed and 100 μL of dimethyl sulfoxide (DMSO, Amresco Inc., Dallas, TX, USA) was added to each well. The absorbance of the solubilized formazan was measured at 595 nm using a spectrofluorometer (F-2500, Hitachi, Tokyo, Japan). The absorbance served as an indicator of cell viability, with it being standardized to cells cultured in the control medium, which were deemed to be 100% alive.

### 2.6. Animals and Treatment

Male ICR mice (23–25 g) were purchased from Chengdu Da Shuo Experimental Animal Co., Ltd. (Chengdu, China). The mice were bred in standard plastic cages with a controlled temperature (22 ± 3 °C) and a 12:12 light–dark cycle, and they were provided with free access to food and water in the animal facility. The experiment was carried out by caring for three animals in a cage dedicated to animals. SJT pretreatment (100 mg/kg) was administered for 7 days, then ISO (30 mg/kg body weight) dissolved in saline was injected subcutaneously for 7 consecutive days. The doses administrated were comparable to those used in mice studies when normalized by body surface area. The control and treatment groups were randomly divided into groups, and the order of randomization was determined by randomization by the researcher with all animals placed in large cages. Control group: fed normal diet and subcutaneous injection of PBS (*n* = 6). ISO group: normal diet and subcutaneous injection of ISO (30 mg/kg·day, *n* = 6). SJT 100 group: normal diet, SJT treatment 100 mg/kg·day, and subcutaneous injection of ISO (*n* = 6). PRO group: normal diet, propranolol treatment 10 mg/kg·day, and subcutaneous injection of ISO (*n* = 6). The number of animals used in the experiment was 24. The ratio of heart weight to body weight was used as an index of cardiac hypertrophy. Mice were anesthetized with 4% isoflurane using an N_2_O & O_2_ flowmeter system (Harvard Apparatus, Small Animal Ventilator, Harvard, MA, USA) mounted on an Anesthesia Tabletop Bracket. The abdominal artery was then incised to sacrifice the animals. All management and use of experimental animals were conducted in accordance with the guidelines of the National Institute of Health and were approved by the Animal Experimental and Utilization Committee of Wonkwang University School of Medicine (approval number: WKU 20-115). Animal cages were repositioned twice a week to minimize potential confounding factors such as treatment and measurement order or animal/cage location. One researcher conducted one analysis, and the experiment was conducted at the same time with the minimum required time. Information on group assignments, etc., at different stages of the experiment (during assignment, conduct of experiment, evaluation of results, and data analysis) was known only to the research director and was not disclosed at the time of analysis. All animals used in the experiment were subjected to the study after a one-week adaptation period. All research procedures were randomized and designed to generate groups of equal size using blinded analysis methods.

### 2.7. Cell Size and Stress Fiber Formation

H9c2 cells were plated in a 6-well plate at a density of 4.5 × 10^5^ cells per well and grown at 37 °C. To assess the full effect of SJT, it was incubated in serum-free media for 14 h before processing. H9c2 cells were treated with SJT (1, 5 g/mL), and 30 min later, they were treated with DOX. After Doxorubicin treatment, they were incubated for 24 h or an appropriate time for each experiment. H9c2 cells were fixed with a 3.7% formaldehyde solution in PBS buffer for 10 min at room temperature. The cells were then permeabilized with 0.1% Triton X-100 for 5 min at room temperature and incubated in PBS containing 1% BSA for 30 min at 37 °C. To visualize F-actin, the cells were stained with phalloidin-Alexa488 (A12379, Thermo Fisher, Waltham, MA, USA) for 20 min at room temperature. Fluorescent images of the fixed samples were acquired using an inverted microscope, specifically the EVOS M5000 Cell Imaging System (Thermo Fisher Scientific, Waltham, MA, USA).

### 2.8. Immunocytochemical Stain

H9c2 cells were cultured in a 6-well plate at a density of 4.5 × 10^5^ cells per well at 37 °C. The cells were incubated in serum-free media for 14 h before processing SJT. The cells were treated with SJT (5 g/mL), and 30 min later, they were treated with Doxorubicin. After Doxorubicin treatment, they were incubated for 24 h. H9c2 cells were fixed with 3.7% formaldehyde solution in PBS buffer for 10 min at room temperature. Cells were permeabilized in 0.1% Triton X-100 for 5 min at RT and incubated in PBS containing 1% BSA for 30 min at 37 °C. Cells were stained with primary antibody overnight at 4 °C to visualize phospho-GATA4 (1:500; sc-377543). After washing, the corresponding secondary antibody was labeled with Alexa Fluor 488 (1:500; Molecular Probes, Eugene, OR) for 1 h at RT. Fluorescent images of fixed samples were acquired on an inverted microscope, using an Eclipse Ti-U inverted microscope (Nikon, Minato, Tokyo, Japan).

### 2.9. RNA Isolation and Real-Time PCR

Total cellular RNA was extracted using TRIzol reagent (Ambion, Carlsbad, CA, USA). cDNA synthesis was performed using the extracted mRNA through a reverse transcription reaction using the SimpliAmp Thermal Cycler (Life Technologies, Carlsbad, CA, USA). The sequences of primers and probes were as follows: ANP (forward: 5′-GCT CGA GCA GAT CGC AAA AG-3′, reverse: 5′-GAG TGG GAG AGG TAA GGC CT-3′), BNP (forward: 5′-AGC CAG TCT CCA GAA CAA TCC A-3′, reverse: 5′-TGT GCC ATC TTG GAA TTT CGA-3′), β-MHC (forward: 5′-CAG AAC ACC AGC CTC ATC AA-3′, reverse: 5′-CCT CTG CGT TCC TAC ACT CC-3′), α-tubulin (forward: 5′-GAC CAA GCG TAC CAT CCA GT-3′, reverse: 5′-CCA CGT ACC AGT GCA CAA AG-3′). Real-Time PCR was conducted using the Step-One Real-Time PCR System, initiating with a denaturation step at 95 °C for 10 min, followed by 40 cycles of 15 s at 95 °C and 60 s at 60 °C (Product No. 4376600, Applied Biosystems, Foster City, CA, USA). Each RNA sample was assessed three times. The resulting mRNA abundance data were normalized against α-tubulin mRNA abundance. Melting curve analysis was performed on PCR products, and the relative mRNA expression was determined using the 2^−ΔΔCt^ method. GAPDH was employed as the internal reference gene, and the mean Ct values were normalized to GAPDH.

### 2.10. Western Blot Analysis

Briefly, an average of 0.13 g of heart tissue was chopped and ground using a glass homogenizer on ice, followed by mixing it with 500 μL of RIPA buffer for 30 min on ice. The solution was then centrifuged at 13,000× *g* for 30 min at 4 °C, and the resulting supernatant was collected. H9C2 cells were cultured in a 100 mm dish at a density of 2.5 × 10^6^ cells per well at 37 °C. They were treated with SJT (5 µg/mL), and 30 min later, they were treated with Doxorubicin. Doxorubicin treatment was followed by a 24 h incubation of the cells or until the necessary expression occurred. Protein lysis was performed using WSE-7420 EzRIPA Lysis buffer (EzWestLumi plus, ATTO Technology, Amherst, New York, NY, USA), supplemented with Protease Inhibitor and Phosphatase Inhibitor. The cell lysate (containing 30 μg of protein) was separated using 10% SDS-polyacrylamide gel electrophoresis (PAGE) and transferred onto a nitrocellulose membrane. The membrane was blocked with 5% BSA powder in TBS-T buffer (10 mM Tris-HCl, pH 7.6, 150 mM NaCl, 0.05% Tween-20) for 1 h, following the supplier’s recommendation. It was then incubated with an appropriate primary antibody in a recommended dilution solution. After washing, the primary antibody was detected using a horseradish peroxidase-conjugated secondary antibody against rabbit IgG (A120-101P) and mouse IgG (A90-116P), and the bands were visualized using an enhanced chemiluminescence system (EzWestLumi plus, ATTO Technology, Amherst, New York, NY, USA). The protein expression levels were determined by analyzing the captured signals on the nitrocellulose membrane using a Chemi-doc image analyzer (iBright FL100, Thermo Fisher Scientific, Waltham, MA, USA).

### 2.11. Preparation of Cytoplasmic and Nuclear Extracts

Nuclear extraction followed the manuals for NE-PER Nuclear and Cytoplasmic Extraction Reagents (Thermo Scientific™, Waltham, MA, USA). For cell culture preparation, adherent cells were harvested using trypsin-EDTA and centrifuged at 500× *g* for 5 min, while suspension cells were collected by centrifugation at 500× *g* for 5 min. After washing with PBS, 1–10 × 10^6^ cells were transferred to a 1.5 mL microcentrifuge tube, pelleted at 500× *g* for 2–3 min, and the supernatant was carefully removed. Then, ice-cold CER I was added to the cell pellet, and the procedure for Cytoplasmic and Nuclear Protein Extraction was followed using the reagent volumes specified in the reagent manual. In this extraction process, the cell pellet was vigorously vortexed, incubated on ice, and mixed with CER II before centrifugation to obtain the cytoplasmic extract. The insoluble pellet fraction containing nuclei was suspended in ice-cold NER, vortexed intermittently, and centrifuged to yield the nuclear extract. Both extracts were stored at −80 °C until further use.

### 2.12. Histopathological Analysis

For histopathological analysis, the hearts were randomly selected from each group and euthanized using isoflurane. Samples were collected from the hearts of other groups and fixed in 10% saline solution for 24 h. The samples were then washed with tap water, dehydrated using a series of alcohol dilutions, cleared in xylene, and embedded in paraffin at 56 °C for 24 h in a hot water oven. Paraffin wax tissue blocks were prepared for sectioning at a thickness of 4 μm using a microtome. The generated tissue sections were collected on glass slides, deparaffinized, stained with hematoxylin and eosin, and examined using an optical microscope (EVOS™ M5000, Thermo Fisher Scientific, Bothell, WA, USA).

### 2.13. Statistical Analysis

All experiments were repeated at least three times and statistical analyses were performed with the *t*-test. Results were expressed as mean ± standard error (S.E.), and data were analyzed using one-way analysis of variance followed by Student’s *t*-test to determine any significant differences. *p* < 0.05 was considered as a statistically significant difference.

## 3. Results

### 3.1. HPLC Fingerprinting Analysis of SJT

**Figure 1:**
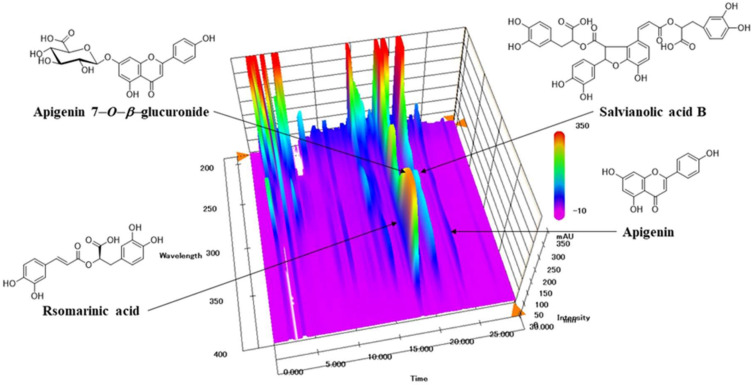
Three-dimensional HPLC chromatogram of SJT.

The typical three-dimensional HPLC chromatogram of SJT is shown in Figure 1. The authentic standards (apigenin, apigenin 7-*O*-β-glucuronide, rosmarinic acid, and salvianolic acid B) were already known to be isolated from each single extract of SJT. Authentication of these four constituents in SJT was performed by comparison of retention time and the specific absorption spectra pattern in between peaks of authentic standards and SJT. The four peaks observed in the SJT chromatogram and its standards apigenin, apigenin 7-*O*-β-glucuronide, rosmarinic acid, and salvianolic acid B perfectly matched in the parallel chromatogram. Moreover, the specific UV absorption spectra pattern of SJT and its four standards showed the same patterns in the 190–400 nm wavelength range with maximum and minimum absorption at those spectral scanning ranges. Therefore, the four major peaks observed in the SJT chromatogram were identified as rosmarinic acid, apigenin 7-*O*-β-glucuronide, salvianolic acid B, and apigenin. The calibration curve was measured in the ranges of 0.63–10.00 (apigenin), 1.25–20.00 μg/mL (apigenin 7-*O*-β-glucuronide), and 2.50–40.00 μg/mL (rosmarinic acid and salvianolic acid B). The calibration curves for rosmarinic acid, apigenin 7-*O*-β-glucuronide, salvianolic acid B, and apigenin were y = 29622.35x − 5060.67, y = 37934.35x − 1671.49, y = 12314.68x − 3764.01, and y = 77610.17x − 2204.89, respectively, and the coefficients of determination were all > 0.9999. Quantification of these four constituents in SJT sample was monitored at 290 nm (salvianolic acid B), 330 nm (rosmarinic acid), and 335 nm (apigenin 7-*O*-β-glucuronide and apigenin). Four constituents (apigenin, apigenin 7-*O*-β-glucuronide, rosmarinic acid, and salvianolic acid B) were detected at concentrations of 1.74, 6.26, 10.71, and 0.38 mg/lyophilized g, respectively.

### 3.2. Effect of SJT on DOX-Induced H9c2 Cell Death

**Figure 2:**
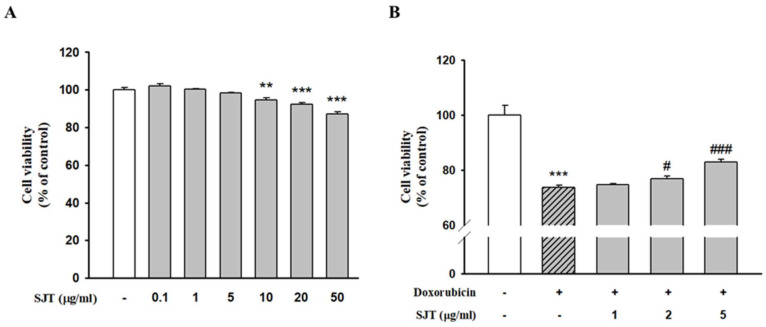
Effect of SJT on DOX–induced H9c2 cell death. (**A**) Cells were treated with concentrations (0–50 μg/mL) of SJT for 24 h. (**B**) H9c2 cells were exposed to doxorubicin (1 μM) while treated with or without SJT at concentrations of 1, 2, and 5 μg/mL for 24 h. Cell viability was measured by Cell Cytotoxicity Assay. The data are expressed as a percentage of basal value and are the means ± S.E of five independent experiments. *** *p* < 0.001, ** *p* < 0.01 vs. control, ### *p* < 0.001, # *p* < 0.05 vs. DOX.

MTT assays were performed to evaluate the cytotoxic effect of SJT on H9c2 cells. As shown in Figure 2, SJT did not alter cell viability at the range of 1–5 μg/mL (>90% cell viability). However, incubation with 10–50 μg/mL SJT significantly decreased cell viability. Thus, SJT was experimented with at a non-cytotoxic concentration (less than 10 μg/mL) in H9c2 cells (Figure 2A). Treatment with DOX for 24 h resulted in decreased cell viability in H9c2 cells. Pretreatment with SJT was observed to alleviate cell damage and cell death (Figure 2). Cell viability was assessed using the MTT assay and normalized to the untreated group, presented as a percentage (the control group was considered 100% cell viability). Elevated cell viability was achieved with a high-dose SJT treatment (*p* < 0.001) compared to the Model (DOX only) group (Figure 2B).

### 3.3. Effect of SJT on DOX-Induced Cardiac Hypertrophy in H9c2 Cells

**Figure 3:**
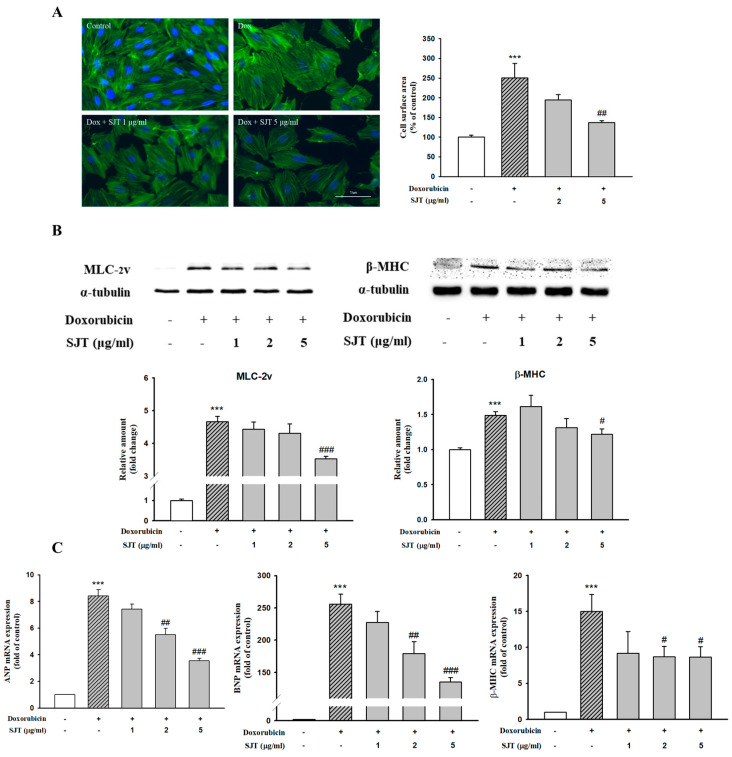
Effect of SJT on DOX–increased cardiac hypertrophy in H9c2 cells. (**A**) H9c2 cells were pretreated with SJT (2, 5 μg/mL) for 30 min and then stimulated with doxorubicin (1 μM) for 24 h. Cells were stained with Alexa Fluor™ 488 Phalloidin F-actin. Cell size was quantified by measuring the surface area of the cells. (**B**) Effect of SJT on DOX–induced hypertrophic protein expression. The protein levels of MLC–2v and β–MHC were determined by Western blot analysis. (**C**) ANP, BNP, and β–MHC mRNA expressions were analyzed using Real–Time PCR. The data are expressed as a percentage of basal value and are the means ± S.E of three independent experiments. *** *p* <0.001 vs. control, ### *p* < 0.001, ## *p* < 0.01, # *p* < 0.05 vs. doxorubicin.

Immunofluorescence assays were performed to determine the effect of SJT on cell stress fiber formation using the antibody against F-actin. The results determined that the DOX-increased cell size was completely abolished by pretreatment with SJT (Figure 3A).

To evaluate the effects of SJT on hypertrophy induced by DOX, H9c2 cells were pretreated for 30 min with 1–5 μg/mL SJT prior to 1 μM DOX exposure. As shown in Figure 3B, MLC-2v and β-MHC protein expressions induced by DOX were inhibited by SJT. In addition, SJT significantly inhibited ANP, BNP, and β-MHC mRNA expressions in DOX-induced H9c2 cells (Figure 3C).

### 3.4. Effect of SJT on DOX-Induced Phosphorylation of p38 and ERK1/2

**Figure 4:**
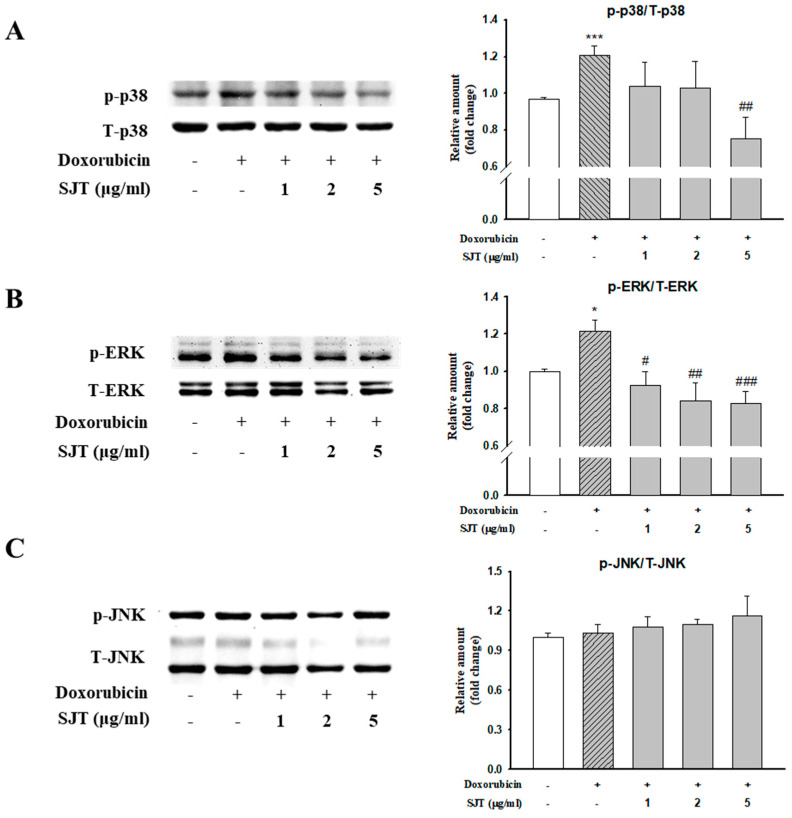
Effect of SJT on DOX–induced phosphorylation of p38 and ERK. H9c2 cells were pretreated with SJT (1–5 μg/mL) for 30 min and then treated with doxorubicin (1 μM) for 1 h. (**A**) p38, (**B**) ERK, and (**C**) JNK MAPK protein expressions were analyzed using Western blot analysis. p38, ERK, and JNK protein levels were used as loading controls. *** *p* < 0.001, * *p* < 0.05 vs. control, ### *p* < 0.001, ## *p* < 0.01, # *p* < 0.05 vs. DOX.

The activation of MAPK signaling pathways is known to be important in cardiac hypertrophy. In this study, we investigated the effect of SJT pretreatment on the activation of MAPK signaling during cardiac hypertrophy, focusing on JNK, ERK1/2, and p38. Among these signaling factors, DOX stimulation led to the activation of phospho-p38. However, pretreatment with SJT completely attenuated the DOX-induced increase in p38 phosphorylation. Similarly, DOX induced the phosphorylation of ERK1/2, but this increase was attenuated by pretreatment (Figure 4A). Stimulation with DOX significantly enhanced the phosphorylation of ERK1/2 in H9c2 cells, and this increase was significantly reduced by pretreatment with SJT (Figure 4B). However, no activation of JNK MAPK was observed following DOX stimulation (Figure 4C).

### 3.5. Effect of SJT on DOX-Induced Calcineurin/NFAT/GATA4 Pathway

**Figure 5:**
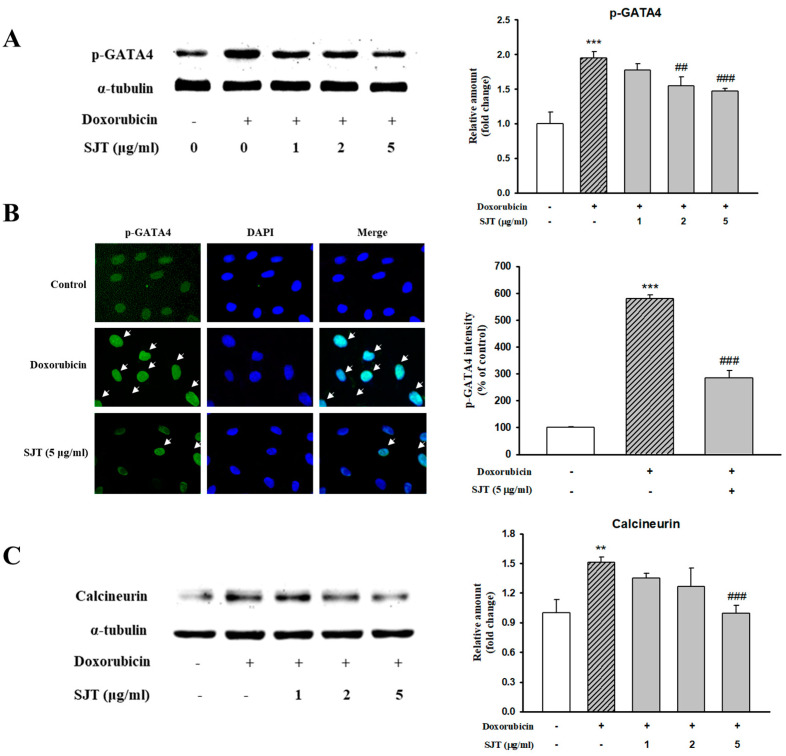
Effect of SJT on DOX–induced GATA–4 and Calcineurin. H9c2 cells were pretreated with SJT (1–5 μg/mL) for 30 min and then treated with DOX (1 μM) for 90 min and 24 h. (**A**) The protein levels of phosphorylated GATA–4 were determined by Western blot analysis. (**B**) Immunofluorescent images of p–GATA–4 nuclear translocation under the laser scanning confocal microscopy are shown (magnification. 400×). Nuclei were stained with DAPI (blue) and p–GATA–4 was stained with Alexa Fluor 488 (green) (immunofluorescence, 200×). (**C**) Calcineurin protein expression was analyzed using Western blot analysis. The results are expressed as the mean ± SE values of three experiments. *** *p* < 0.001, ** *p* < 0.01 vs. control, ### *p* < 0.001, ## *p* < 0.01 vs. DOX.

GATA4, an important transcription factor related to cardiac hypertrophy, promotes cardiac hypertrophic marker proteins including ANP, BNP, and β-MHC. This result determined whether DOX-induced hypertrophy was associated with p-GATA4 using Western blot analysis and immunostaining. SJT inhibited the DOX-induced phosphorylation protein expression of GATA4 in H9c2 cells (Figure 5A). To confirm the consistency with the results of the Western blot analysis, immunofluorescence staining was performed using the phosphor-GATA4 antibody. Nuclei were stained with DAPI (blue) and p-GATA was stained with Alexa Fluor 488. As a result, DOX increased p-GATA4 phosphorylation in the nucleus, but phosphorylation was significantly decreased in the case of treatment with SJT (Figure 5B). In addition, SJT inhibited DOX-induced calcineurin expression in a dose-dependent manner (Figure 5C).

**Figure 6:**
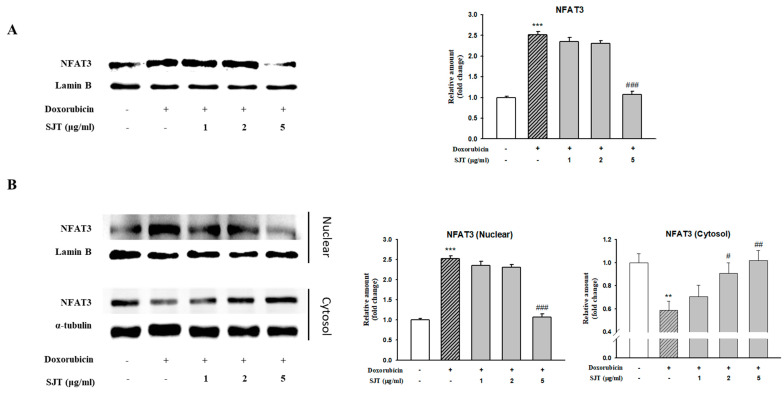
Effect of SJT on DOX–induced NFAT expression. H9c2 cells were pretreated with SJT (1–5 μg/mL) for 30 min and then treated with DOX (1 μM) for 90 min and 24 h. (**A**) NFAT–3 protein expressions were analyzed using Western blot analysis. (**B**) Effect of SJT on nuclear translocation of NFAT–3 was confirmed in H9c2 cells exposed to DOX. The results are expressed as the mean ± SE values of three experiments. *** *p* < 0.001, ** *p* < 0.01 vs. control, ### *p* < 0.001, ## *p* < 0.01, # *p* < 0.05 vs. DOX.

As shown in Figure 6A, DOX enhanced calcineurin protein expression, whereas it was inhibited by SJT. In addition, NFAT3 levels were also enhanced in DOX-induced H9c2 cells. However, this elevation was significantly attenuated by SJT. Therefore, these results suggest that SJT improves cardiac hypertrophy by regulating the calcineurin/NFAT/GATA4 pathway.

### 3.6. Effect of SJT on DOX-Induced Cardiac Apoptosis

**Figure 7:**
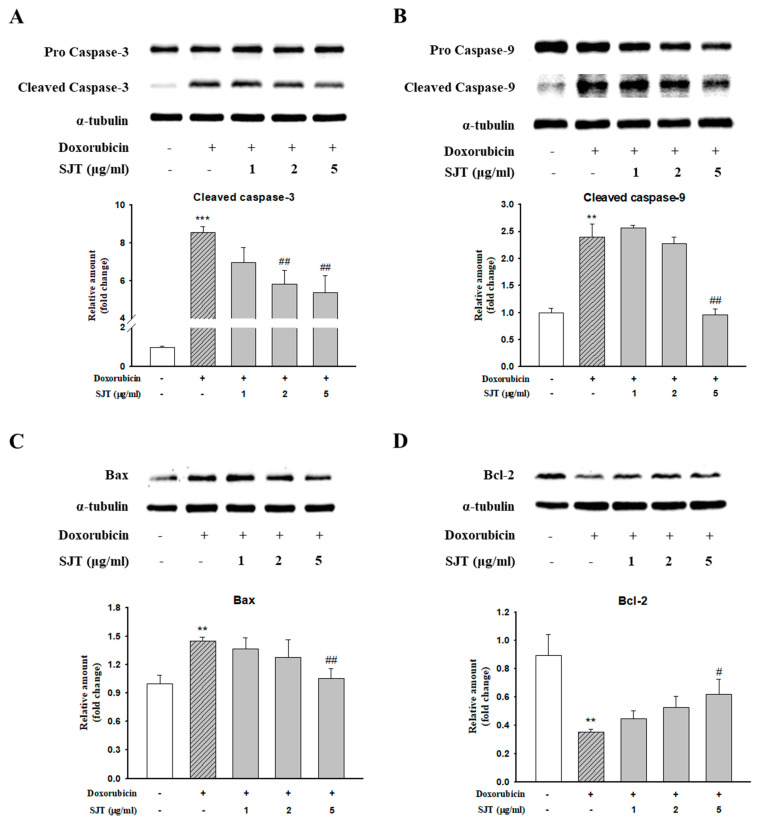
Effect of SJT on DOX–induced cardiac apoptosis. H9c2 cells were pretreated with SJT (1–5 μg/mL) for 30 min and then treated with DOX (1 μM) for 18 h and 12 h. (**A**) Caspase–3, (**B**) Caspase–9, (**C**) Bax, and (**D**) Bcl–2 protein expressions were analyzed using Western blot analysis. The results are expressed as the mean ± SE values of three experiments. *** *p* < 0.001, ** *p* < 0.01 vs. control, ## *p* < 0.01, # *p* < 0.05 vs. doxorubicin.

To clarify the effect of SJT on apoptosis in H9c2 cells exposed to DOX, we performed Western blot analysis in H9c2 cells. DOX induced cleaved caspase-3, cleaved caspase-9, and Bax protein expression, whereas SJT inhibited DOX-induced cleaved caspase-3, cleaved caspase-9, and Bax protein expression (Figure 7A–C). In addition, DOX decreased Bcl-2 protein expression, whereas pretreatment of SJT increased DOX-inhibited Bcl-2 protein expression (Figure 7D). Based on these results, it can be inferred that SJT may improve cardiac hypertrophy-induced heart failure by enhancing the attenuation of DOX-induced cardiac apoptosis through the modulation of apoptosis-related factors.

### 3.7. The Effect of SJT on Cardiac Hypertrophy in ISO-Treated Mice

**Figure 8:**
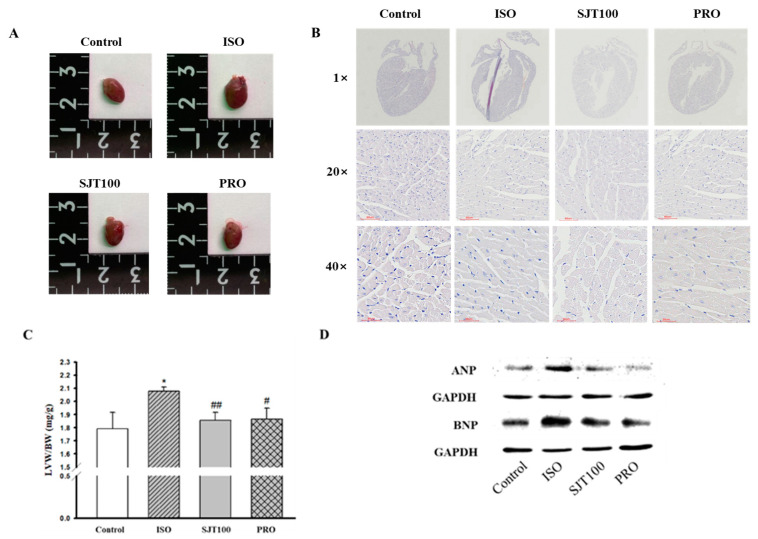
Effect of SJT on ISO–induced cardiac hypertrophy. Effect of SJT on heart size (**A**) and left ventricular/body weight (**B**) in ISO–induced ICR mice. (**C**) Images of the whole hearts of animal models. (**D**) Effect of SJT on cardiomyocyte hypertrophy markers in left ventricular tissues. Mice were infused with PBS (control), ISO, propranolol 30 mg/kg·day with ISO (PRO), and SJT 100 mg/kg·day with ISO (SJT–100). Data are expressed as mean ± SE. There are 6 experimental cases. * *p* < 0.05 vs. Control, ## *p* < 0.01, # *p* < 0.05 vs. ISO. BW, body weight; LVW, left ventricular weight; Iso, isoprenaline; PRO, propranolol.

To identify whether SJT has a protective effect on cardiac hypertrophy, we pretreated mice with SJT (100 mg/kg/day) and then co-administered ISO (30 mg/kg/day). As shown in Figure 8A,C, the SJT group had reduced heart size and left ventricle/body weight ratio compared to the control group (*p* < 0.05). In addition, heart morphology was also ameliorated by administration of SJT (Figure 8B). We next examined the effects of TGW on the expression of ANP and BNP, which are markers of cardiac hypertrophy. As shown in Figure 8D, in the ISO group, there was a significant increase in the protein expression of cardiac hypertrophic markers in the left ventricle tissue. The SJT group had significantly reduced ISO-increased ANP and BNP protein expression. These results showed that administration of SJT (100 mg/kg/day) inhibited cardiac hypertrophy in ISO-treated mice.

## 4. Discussion

Cardiac hypertrophy develops as an adaptive response to various diseases such as myocardial infarction and hypertension and leads to deterioration of cardiac function and chronic heart failure [3,4]. SJT has anti-tumor, anti-inflammatory, and anti-allergy effects [43]. Among the compounds of SJT, salvianolic acid B, rosmarinic acid, and apigenin attenuate cardiac hypertrophy, ischemia, myocardial infarction, and arrhythmias [30,45,46]. However, the effect of SJT in cardiac hypertrophy has been unclear. Therefore, this study has demonstrated for the first time that SJT blocks the cardiac hypertrophy induced by DOX, through the inhibition of the Calcineurin/NFAT/GATA4 pathway.

Stress fibers, commonly known as F-actin and α-actinin, are primarily composed of bundled actin filaments and serve as the contractile system based on actomyosin in cells [15]. Dysregulation of stress fiber formation is commonly observed in nearly all major cardiovascular disorders, including hypertension, myocardial syndrome, heart failure, and cardiac remodeling after myocardial infarction [17]. In the present study, immunofluorescence staining revealed that pretreatment with SJT reduced the DOX-induced cell surface area by decreasing stress fiber formation (F-actin). A previous study has shown that cardiac hypertrophy was attenuated by reducing stress fiber formation [18]. Therefore, these results suggest that SJT decreases cell surface area through attenuating stress fiber formation.

The release of ANP and BNP, cardiac hypertrophic biomarkers, occurs during the development of cardiac hypertrophy [47]. In addition, one of the typical characteristics of cardiac hypertrophy is an increase in the expression of β-MHC and MLC-2v [48,49]. This result was examined to find out whether SJT has an inhibitory effect on the mRNA expression of ANP, BNP, and β-MHC as well as the protein expression of β-MHC and MLC-2v. These results showed that pretreatment with SJT reduced the expression of ANP and BNP in DOX-treated H9c2 cells. In addition, the DOX-induced expression of β-MHC and MLC-2v was inhibited by pretreatment with SJT. The results of this study demonstrated that SJT has an inhibitory effect on the DOX-induced expression of ANP, BNP, β-MHC, and MLC-2v in H9c2 cells. These results suggest that SJT may have a potential role in cardiac hypertrophy.

Evidence has suggested that the MAPK family including ERK1/2, p38, and JNK plays pivotal roles in the development of cardiac hypertrophy [50,51]. This result showed that SJT significantly inhibited the DOX-induced phosphorylation of p38 and ERK1/2. A previous study has shown that the ERK1/2-GATA4 and p38-GATA4 pathways induced by Angiotensin II are associated with cardiac hypertrophy in neonatal rat cardiomyocytes [52]. Thus, to investigate the anti-hypertrophic effect of SJT on DOX-treated H9c2 cells, this study examined the protein expression of p-GATA4. The results of this study revealed that SJT reduced the DOX-induced phosphorylation of GATA4, which has been identified to have an important role in the transcription of the hypertrophic gene. These results suggest that SJT alleviates cardiac hypertrophy by inhibiting the DOX-induced MAPK (p38 and ERK1/2)-GAPA4 pathway.

The Calcineurin/NFAT pathway is one of the key signaling pathways associated with cardiac hypertrophy. Calcineurin, serine-threonine phosphatase, promotes dephosphorylation and translocation of the nuclear factor of activated T cells (NFAT) to the nucleus, leading to cardiac hypertrophy [36]. This study sought to find out whether SJT modulates the expression of Calcineurin and NFAT3 induced by DOX. These data showed that SJT inhibited the DOX-induced expression of Calcineurin and NFAT3 in H9c2 cells. These results suggest that SJT may have a significant protective effect against cardiac hypertrophy by regulation of the calcineurin/NFAT3/GATA4 signaling pathway.

As hypertrophic stimuli persist, cardiomyocytes undergo a loss of cell viability and the disruption of cardiac structure, ultimately leading to heart failure [53]. Numerous experimental studies have revealed that the activation of caspases is a molecular characteristic of apoptosis and they play a crucial regulatory role in the apoptotic cascade [54]. Caspase-9 acts as the initiating enzyme in the mitochondrial apoptotic pathway, while caspase-3 serves as the key effector caspase, which is activated through cleavage by caspase-9 [55]. Thus, this result was performed to determine the expression of the cleaved form of caspase-9 and caspase-3, which is the active form in H9c2 cells. The protein expression of cleaved caspase-9 and cleaved caspase-3 induced by DOX was significantly reversed by pretreatment with SJT. These results indicate that SJT may have a significant protective effect against cardiac hypertrophy by regulating the apoptosis signaling pathway.

This study indicated that SJT attenuated DOX-induced stress fiber formation and regulated the DOX-enhanced MAPK-GATA4, calcineurin-NFAT3, and apoptosis pathway. In addition, SJT alleviated the expression of cardiac hypertrophic biomarkers, the ANP, BNP, β-MHC, and MLC-2v levels induced by the hypertrophy model. In conclusion, these data provide the first piece of evidence that SJT may play an important role in the prevention of cardiac hypertrophy and apoptosis. The findings imply that SJT could be a promising intervention for cardiac hypertrophy and its associated heart failure. The protective effects of SJT, as a traditional herbal medicine, offer valuable insights for the development of novel therapeutic strategies in managing heart failure.

## Data Availability

Data are available on reasonable request.
